# Supplementary material for: FERN – a Java framework for stochastic simulation and evaluation of reaction networks
Source: BMC Bioinformatics. 2008 Aug 29;9:356. doi: 10.1186/1471-2105-9-356 (PMC2553347; doi:10.1186/1471-2105-9-356)
Supplement: Additional file 1 — FERN distribution, Version 1.3. This archive contains the FERN source code and binaries as well as documentation and example models in FernML and SBML. [file 1471-2105-9-356-S1.zip › fern/doc/javadoc/fern/simulation/observer/package-use.html]

Uses of Package fern.simulation.observer


---


|  |  |  |
| --- | --- | --- |
| **Overview** \| **Package** \| Class \| **Use** \| **Tree** \| **Deprecated** \| **Index** \| **Help** | |  |
| PREV   NEXT | **FRAMES**    **NO FRAMES**     **All Classes** |  |


---


## **Uses of Package fern.simulation.observer**

| Packages that use fern.simulation.observer | |
| --- | --- |
| **fern.cytoscape** | Provides the classes for the cytoscape plugin. |
| **fern.example** | Contains examples and demonstrations of the framework. |
| **fern.network.sbml** | Provides the classes for parsing and using sbml based networks. |
| **fern.simulation** | Provides algorithms for simulating reaction network as well as methods for observer certain aspects of a simulation. |
| **fern.simulation.observer** | Provides classes to observe certain aspects of simulations. |

Classes in fern.simulation.observer used by fern.cytoscape

****Observer****
  
          Implementing classes are used to observe certain aspects of simulations.

Classes in fern.simulation.observer used by fern.example

****Observer****
  
          Implementing classes are used to observe certain aspects of simulations.

Classes in fern.simulation.observer used by fern.network.sbml

****Observer****
  
          Implementing classes are used to observe certain aspects of simulations.

****TriggerObserver****

Classes in fern.simulation.observer used by fern.simulation

****Observer****
  
          Implementing classes are used to observe certain aspects of simulations.

Classes in fern.simulation.observer used by fern.simulation.observer

****GnuPlotObserver****
  
          Interface for certain observer which can produce gnuplot data.

****IntervalObserver****
  
          Base class for observing certain aspects repeatedly after a given interval.

****Observer****
  
          Implementing classes are used to observe certain aspects of simulations.

---


|  |  |  |
| --- | --- | --- |
| **Overview** \| **Package** \| Class \| **Use** \| **Tree** \| **Deprecated** \| **Index** \| **Help** | |  |
| PREV   NEXT | **FRAMES**    **NO FRAMES**     **All Classes** |  |


---
